# A study on the impact of perceived teacher emotional support on university students’ online learning engagement: the mediating role of academic burnout

**DOI:** 10.3389/fpsyg.2025.1625857

**Published:** 2025-08-05

**Authors:** Lina Sun, Rongshuang Ma, Chaohui Du, Jianmin Zhao, Yuliu Yang, Xiaoguang Zhang

**Affiliations:** ^1^School of Computer and Information Technology, Northeast Petroleum University, Daqing, China; ^2^Chuang Ke Primary School, Dalian, China; ^3^School of Educational Science, Harbin Normal University, Harbin, China; ^4^School of Mechanical Science and Engineering, Northeast Petroleum University, Daqing, China

**Keywords:** perceived teacher emotional support, academic burnout, online learning, learning engagement, university students

## Abstract

**Introduction:**

This study investigates the impact of perceived teacher emotional support on university students’ engagement in online learning, with a particular focus on the mediating role of academic burnout. Although prior research has established that perceived teacher emotional support positively influences learning engagement and that academic burnout has a negative effect, the underlying mechanisms among these three variables in an online learning context remain unclear.

**Methods:**

Participants were drawn from a public university in China. Data were collected from 361 undergraduate students through a structured questionnaire, and the relationships among the key variables were analyzed using Structural Equation Modeling (SEM).

**Results:**

The findings indicate that perceived teacher emotional support is a significant predictor of online learning engagement. Moreover, academic burnout serves as a mediator: higher levels of perceived emotional support from teachers are associated with lower levels of burnout and increased engagement in online learning activities. These results underscore the critical role of perceived teacher emotional support in mitigating academic burnout and enhancing students’ motivation and participation in online learning environments.

## 1 Introduction

The rapid growth of internet technology in the digital age has significantly transformed the field of education. Online learning has gradually become a crucial component of modern education, providing learners with unprecedented opportunities and experiences (Dhawan, 2020). However, online learning environments also present significant challenges, such as the lack of emotional connection between students and instructors, as well as lower student engagement compared to traditional face-to-face learning (Xie et al., 2020; Martin and Borup, 2022). Learning engagement is a reliable indicator of online learning quality (Phan et al., 2016). Therefore, understanding the factors that affect online learning engagement is crucial importance. And many studies have focused on identifying these key factors.

Online learning engagement refers to the sustained focus and involvement students demonstrate while using online learning platforms. Scholars generally categorize online learning engagement into three dimensions: cognitive, emotional, and behavioral (Hu et al., 2016). The factors influencing online learning engagement are numerous and complex. Most studies treat online learning engagement as the outcome variable and examine how other factors influence it. From the student’s perspective, several factors affect engagement, including the willingness to use electronic devices, self-efficacy (Getenet et al., 2024), motivation and attitude (Ferrer et al., 2022), and personality traits (Yan et al., 2024). From the teacher’s perspective, existing research has confirmed the impact of teacher support on learning engagement (Ma et al., 2023; Wang L, 2022; Luan et al., 2023), including aspects such as teacher-student interaction (Sun et al., 2022) and feedback (Pan and Shao, 2020). In addition to these factors, the learning environment (Luo et al., 2022) and parental support have also been identified as significant contributors to online learning engagement (Novianti and Garzia, 2020).

The concept of “burnout” originally derives from “occupational burnout” which refers to the phenomenon of individuals experiencing fatigue, depression, and other negative emotions due to prolonged exposure to stress (Freudenberger, 1974). In the context of education, this is known as academic burnout. Academic burnout is characterized by students’ feelings of boredom, frustration, and a lack of motivation or interest in learning, yet still feel the obligation to engage in it. This often leads to avoidance behaviors that hinder their learning (Yang and Lian, 2005). In online learning environments, the lack of emotional connections between students and instructors, as well as among students themselves, contributes to higher levels of academic burnout. Furthermore, increased screen time and prolonged use of electronic devices can exacerbate stress and contribute to burnout (Mheidly et al., 2020; Mu and Guo, 2022).

Existing research has shown that teachers’ emotional support plays a mitigating and buffering role in reducing academic burnout and other negative behaviors (Karimi and Fallah, 2021). However, it remains unclear whether university students truly perceive this support and whether it effectively alleviates their negative behaviors. This issue requires further investigation. Moreover, a review of the relevant literature also indicates that teacher emotional support positively affects learning engagement (Yan et al., 2024), while academic burnout negatively affects it (Mu and Guo, 2022). However, most previous studies have focused on face-to-face learning environments and have been limited to examining the relationships between two variables at a time. To date, no study has integrated teacher emotional support, academic burnout, and learning engagement into a single comprehensive framework for analysis. Therefore, several critical questions remain: How do university students perceive teacher emotional support in an online learning environment? What impact does this support have on learning engagement? And what role does academic burnout play in the relationship between perceived teacher emotional support and learning engagement?

This study examines the effect of perceived teacher emotional support on online learning engagement and tests the mediating role of academic burnout. It aims to expand the theoretical understanding of online learning engagement and clarify how perceived teacher emotional support influences this process. By focusing on perceived teacher emotional support, the study provides practical recommendations for preventing and intervening academic burnout, with the goal of enhancing university students’ online learning engagement.

## 2 Theoretical background and hypotheses

### 2.1 Perceived teacher emotional support

Yeung and Leadbeater (2010) argue that teachers’ encouragement, respect, and other forms of emotional support exhibited during the teaching process play a crucial role (Yeung and Leadbeater, 2010). Once students perceive this emotional support, it can have a positive impact on their learning (He et al., 2024). Therefore, some scholars focus more on students’ perceptions. For instance, scholars such as Gao et al. (2017) emphasize the importance of students’ perceived care, affection, respect, support, and assistance from teachers (Gao et al., 2017). This study adopts a learner-centered perspective and defines teacher emotional support as students’ perceived respect, care, and understanding from their teachers (Ryan and Patrick, 2001). Previous studies have found that such support contributes to the establishment of positive teacher-student relationships, enhances students’ positive emotions, fosters academic autonomy, and significantly improves academic achievement (Guo et al., 2023).

### 2.2 The effect of perceived teacher emotional support on learning engagement

Teacher emotional support is categorized under the emotional support dimension of social support, constituting a crucial component within the social support system. According to Maslow’s hierarchy of needs theory, every individual has a fundamental need for belonging and love, with emotional support being the most direct way to fulfill this need (McLeod, 2007). Teacher emotional support helps meet students’ psychological needs, thereby positively influencing their learning engagement (Jin and Wang, 2019). Previous studies have shown a significant positive relationship between perceived teacher emotional support and online learning engagement (Yan et al., 2024). When students perceive emotional support from their teachers, it enhances their learning engagement (Wang et al., 2022), especially in terms of cognitive and affective engagement (Liao et al., 2023). Research indicates that teacher care and attention enhance both emotional and behavioral engagement, regardless of gender (Bru et al., 2019). Additionally, teacher emotional support indirectly boosts students’ online academic engagement by enhancing their intrinsic motivation (Wang Y., 2022). Existing research suggests that the more emotional support students receive from their teachers, the greater their behavioral, affective, and cognitive engagement in online learning. Therefore, based on Maslow’s theory and previous research, this study proposes the following hypothesis:

*H1. Perceived teacher emotional support positively and significantly predicts university students’ online learning engagement.*

### 2.3 The impact of academic burnout on online learning engagement

Academic burnout is prevalent among university students and can affect their learning engagement to varying degrees (Cano et al., 2024; Nurani et al., 2022). In online learning environments, the impact of academic burnout is particularly significant. Existing research has demonstrated a significant negative correlation between online learning engagement and academic burnout (Cazan, 2015). The online learning environment provides an easy escape for students experiencing high levels of academic burnout, leading to reduced time spent on learning engagement (Wang and Cai, 2021). Additionally, studies have found that academic burnout during online learning is not only negatively correlated with students’ cognitive engagement but also tends to increase over time (Huang et al., 2024). Based on these findings, the following hypothesis is proposed:

*H2. Academic burnout negatively predicts online learning engagement among university students.*

### 2.4 The effect of perceived teacher emotional support on academic burnout

Existing research has shown a significant negative correlation between perceived teacher emotional support and academic burnout (Karimi and Fallah, 2021). Romano et al. (2020) found that high school students’ perception of teacher emotional support is a strong predictor of lower academic burnout (Romano et al., 2020). Similarly, Li et al. (2019) confirmed that middle school students who perceive more emotional support from their teachers tend to feel more confident in their learning and experience less academic burnout (Li et al., 2019). Additionally, research indicated that middle school students with higher levels of perceived teacher emotional support experience lower academic burnout (An, 2020). In online learning environments, teacher emotional support plays a crucial role in alleviating academic burnout caused by the lack of face-to-face interaction and the physical and temporal separation between teachers and students (Yang et al., 2022). Overall, both domestic and international studies have consistently found that perceived teacher emotional support helps alleviate and inhibit academic burnout. Based on these findings, this study proposes the following hypothesis:

*H3. Perceived teacher emotional support negatively predicts academic burnout among university students.*

## 3 Materials and methods

### 3.1 Participants

This study involved 361 first-year students from a university in Daqing, China. The study was conducted entirely in an online environment, and all participants were fully informed of the research objectives before data collection This study focused on first-year university students, who had undergone two and a half years of online learning during their high school education, thereby possessing more profound experiences and perceptions of online learning. The final sample consisted of 224 responses, among which 213 were valid, resulting in a validity rate of 95%. Among the valid responses, 164 were male (77%) and 49 were female (23%), all of whom were enrolled in science-related majors.

### 3.2 Instruments

The scales used in this study were adapted from well-established domestic and international scales, and modified based on teaching practices. A five-point Likert scale was used to measure the variables, ranging from 1 (strongly disagree) to 5 (strongly agree). In the preliminary survey, we collected 126 valid questionnaires. Through item analysis and exploratory factor analysis, the scales were examined, and a total of 14 substandard items were removed across the following three scales. The wording of the items was adjusted to better align with the learners’ context.

#### 3.2.1 Perceived teacher emotional support scale

This study primarily referenced the “Middle School Students’ Perception of Teacher Emotional Support Scale” (Gao et al., 2017). To better align with the online learning context, the perceived teacher emotional support scale was created, drawing from relevant existing scales. The final scale consisted of 15 items, categorized into three dimensions: respect, care, and understanding.

The respect dimension includes 4 items, adapted from Gao et al. (2017) and Xu et al. (2014), such as “During online learning, the teacher listens to and understands my suggestions or views before offering their own, which helps me engage more in learning.”

The care dimension includes 7 items, adapted from Gao et al. (2017), Johnson et al. (1983), Sakiz et al. (2012), and Overall et al. (2011), including statements such as “During online learning, the teacher acknowledges my progress (e.g., praising improvements in my assignment completion or work quality), which encourages me to engage more in learning.”

The understanding dimension consists of 4 items, adapted from Gao et al. (2017), Xu et al. (2014), and Song et al. (2015), including statements such as “During online learning, the teacher understands my difficulties (e.g., needing help with software downloads or needing more time to complete assignments), which motivates me to engage more in learning.”

The internal consistency coefficients for the respect, care, and understanding dimensions were 0.886, 0.933, and 0.924, respectively, while the overall reliability of the scale was 0.955. After revision, the model fit results for the perceived teacher emotional support scale showed CMIN/DF = 2.150 (<3), GFI = 0.926 (>0.90), CFI = 0.957 (>0.90), NFI = 0.923 (>0.90), IFI = 0.957 (>0.90), SRMR = 0.048 (<0.08), and RMSEA = 0.074 (<0.08), all meeting the established standards.

#### 3.2.2 Academic burnout scale

This study adapted the “College Student Learning Burnout Scale” developed by Lian et al. (2005) and made necessary revisions to create a learning burnout scale. The final version consists of 14 items, divided into three dimensions: depressed mood (6 items), inappropriate behavior (5 items), and low sense of achievement (3 items). The consistency coefficients for the three dimensions—depressed mood, inappropriate behavior, and low sense of achievement—were 0.900, 0.842, and 0.855, respectively, while the overall reliability of the scale was 0.911. After revision, the model fit results for the academic burnout scale were as follows: CMIN/DF = 2.303 (<3), GFI = 0.909 (>0.90), CFI = 0.959 (>0.90), NFI = 0.931 (>0.90), IFI = 0.960 (>0.90), SRMR = 0.044 (<0.08), and RMSEA = 0.077 (<0.08), all of which meet the acceptable standards.

#### 3.2.3 Online learning engagement scale

This study adapted the “Online Learning Engagement Scale” from established international scales, making necessary revisions. The final scale consists of 17 items, categorized into three dimensions: behavioral engagement (8 items), cognitive engagement (5 items), and affective engagement (4 items).

The behavioral engagement dimension includes 8 items, adapted from Sun and Rueda (2012) and Deng et al. (2020), with additional self-developed items such as “During online learning, I actively answer the teacher’s questions” and “When I do not understand a certain concept during online learning, I will ask the teacher to explain it again (e.g., using the platform’s hand-raise function or sending a real-time comment, etc.).” The cognitive engagement dimension consists of 5 items, adapted from Sun and Rueda (2012). The affective engagement dimension includes 4 items, also adapted from Sun and Rueda (2012), with additional self-developed items, such as “Online learning sometimes makes me feel sleepy.,” “During online learning, I find it very boring.,” and “I dislike online assessments.”

The consistency coefficients for behavioral, cognitive, and affective engagement, as well as the overall scale consistency, were 0.909, 0.850, 0.859, and 0.916, respectively. The model fit indices of the revised Online Learning Engagement Scale were as follows: CMIN/DF = 1.792 (<3), GFI = 0.907 (>0.90), CFI = 0.954 (>0.90), NFI = 0.903 (>0.90), IFI = 0.955 (>0.90), SRMR = 0.047 (<0.08), and RMSEA = 0.061 (<0.08), all of which meet the acceptable standards.

### 3.3 Common method bias test

To avoid the issue of common method bias, this study employed anonymous scale completion and reverse scoring in the distribution and processing of the scales. However, to further verify the presence of serious common method bias, Har-man’s single-factor test was conducted (Zhou and Long, 2004).

Using SPSS 25.0, an exploratory factor analysis was performed on all items from the Online Learning Engagement Scale, Academic Burnout Scale, and Perceived Teacher Emotional Support Scale. Eight factors with eigenvalues greater than 1 were extracted, with the first factor accounting for 33.074% of the variance, which is below the 40% threshold (Deng et al., 2018). This indicates that common method bias is not a significant concern in this study, allowing for further analysis.

## 4 Data analysis and results

The statistical analysis in this study was primarily conducted using IBM SPSS Statistics 25.0. First, confirmatory factor analysis (CFA) was performed to assess the validity of the scales, and reliability was examined using Cronbach’s alpha. Subsequently, descriptive statistics and correlation analyses were conducted. Based on the theoretical framework and research hypotheses, a structural equation model (SEM) was constructed and analyzed using Amos 24.0 to evaluate model fit. This analysis aimed to explore the relationship between perceived teacher emotional support and university students’ online learning engagement, specifically examining the mediating role of academic burnout.

### 4.1 Measurement model analysis

The Cronbach’s alpha values for each scale and its dimensions were calculated, and confirmatory factor analysis (CFA) was conducted to assess the reliability and convergent validity of the measurement model. To determine whether the model exhibits discriminant validity, chi-square difference tests were performed between different models (Wu, 2010).

Using AMOS 24.0, the dimensions of the structural equation models for the online learning engagement, academic burnout, and perceived teacher emotional support were combined to form a three-factor model (original model), a two-factor model, and a single-factor model. Model fit indices were examined and compared across these models. The results are presented in the Tables 1, 2.

**Table 1:** Reliability and convergent validity.

Instrument	DIM	Composite reliability	Convergence validity
		CR	AVE
Perceived teacher emotional support	RS	0.879	0.646
	CS	0.914	0.605
	US	0.723	0.914
Academic burnout	DM	0.893	0.585
	IB	0.819	0.602
	LSA	0.839	0.635
Online learning engagement	BE	0.858	0.433
	AE	0.833	0.562
	CE	0.857	0.548

RS—respect support. CS—care support. US—understanding support. DM—depressed mood. IB—inappropriate behavior. LSA—low sense of achievement. BE—behavioral engagement. AE—affective engagement. CE—cognitive engagement.

**Table 2:** Discriminant validity results.

Scale	Model	χ^2^	df	CFI	NFI	RMSEA	Results of model comparison tests		
							Model comparison	∆χ^2^	∆df
Online Learning Engagement	Three-factor model (original model)	154.750	103	0.972	0.922	0.049			
	Two-factor model 1	309.980	105	0.888	0.843	0.096	2vs1	155.230***	2
	Two-factor model 2	386.040	105	0.847	0.804	0.112	3vs1	231.290***	2
	Single-factor model	451.840	106	0.812	0.771	0.124	4vs1	297.090***	3
Perceived Teacher Emotional Support	Three-factor model (original model)	179.650	78	0.959	0.931	0.078			
	Two-factor model 1	261.142	80	0.928	0.900	0.103	2vs1	81.492***	2
	Two-factor model 2	330.359	80	0.900	0.873	0.121	3vs1	150.709***	2
	Single-factor model	398.029	81	0.873	0.847	0.136	4vs1	218.379***	3
Academic Burnout	Three-factor model (original model)	109.670	51	0.957	0.923	0.074			
	Two-factor model 1	207.580	53	0.886	0.854	0.117	2vs1	97.910***	2
	Two-factor model 2	310.400	53	0.810	0.782	0.151	3vs1	200.730***	2
	Single-factor model	402.789	54	0.743	0.717	0.175	4vs1	293.119***	3

χ^2^—chi-square test of model fit. df—degrees of freedom. RMSEA—root mean square error of approximation. CFI—comparative fit index. NFI—normalized fit index. Correlations are significant at the *** 0.001 level (two-tailed).

The Cronbach’s alpha values all exceed 0.8, and the composite reliability (CR) values are greater than 0.7, indicating good internal consistency reliability of the measurement scales (Wu, 2010). All standardized factor loadings are above 0.7, and the Average Variance Extracted (AVE) values for affective engagement and cognitive engagement were greater than 0.5, indicating effective reflection of the latent variables. The AVE value for behavioral engagement was 0.433, which falls between 0.4 and 0.5. According to Fornell and Larcker (1981), the AVE value is a relatively conservative estimate of measurement model validity (Fornell and Larcker, 1981). Considering that the CR value was well above the recommended threshold, indicating acceptable internal reliability of the measurement items, an AVE value slightly below 0.5 is deemed acceptable (Lam, 2012).

Additionally, the model fit indices of the alternative models formed by recombining the scale dimensions were inferior to those of the original model, and all models passed the significance test (*p* < 0.001). This confirms that the Perceived Teacher Emotional Support, Online Learning Engagement, and Academic Burnout scales exhibit sufficient discriminant validity. Collectively, these analyses demonstrate that our measurement model exhibits good reliability, convergent validity, and discriminant validity.

### 4.2 Descriptive statistical analysis

Descriptive statistics and correlation coefficients for each variable are shown in Table 3. Academic burnout (M = 3.256, SD = 0.758) and online learning engagement (M = 3.324, SD = 0.484) were both at moderate to high levels, while perceived teacher emotional support (M = 4.028, SD = 0.687) was relatively high. Perceived teacher emotional support was significantly positively correlated with online learning engagement and significantly negatively correlated with academic burnout. In addition, academic burnout was also significantly negatively correlated with online learning engagement. These findings suggest that perceived perceived teacher emotional support strongly influences both online learning engagement and academic burnout. Furthermore, gender showed no significant correlation with academic burnout, online learning engagement, and perceived teacher emotional support. Therefore, the potential impact of gender is excluded from the subsequent analysis.

**Table 3:** Correlation of variables.

Variables	M	SD	1	2	3	4
1. Academic Burnout	3.265	0.758	1			
2. Online Learning Engagement	3.324	0.484	−0.386**	1		
3. Perceived Teacher Emotional Support	4.028	0.687	−0.266**	0.425**	1	
4. Gender	-	-	−0.505	1.747	−0.327	1

Gender is a dummy variable, male = 1, female = 2. Correlations are significant at the ** 0.01 level (two-tailed).

### 4.3 The impact of perceived teacher emotional support on university students’ online learning engagement: the mediating role of academic burnout

The study employs perceived teacher emotional support as the independent variable, online learning engagement as the dependent variable, academic burnout as the mediating variable. A structural equation model was constructed using Amos 24.0, and the Bootstrap method was applied to further examine the mediating effect of academic burnout. The resampling process was repeated 5,000 times to calculate the 95% confidence intervals for both the Bias-Corrected and Percentile methods. If the 95% confidence interval does not include zero, the effect is considered significant (Taylor et al., 2008). The path effect values between variables were estimated using the Maximum Likelihood (ML) method with the Bootstrap approach. The effect values among the variables are presented in Table 4.

**Table 4:** Analysis of mediating effects between perceived teacher emotional support, academic burnout, and online learning engagement.

Effect	Pathways	95% confidence intervals	Effect value (standardized path factor)	Significance
Intermediary effect	Perceived Teacher Emotional Support → Academic Burnout → Online Learning Engagement	[0.102, 0.489]	0.281	***
Direct effect	Perceived Teacher Emotional Support → Online Learning Engagement	[0.215, 0.631]	0.407	***
Total effect		[0.515, 0.898]	0.688	***

Correlations are significant at the *** 0.001 level (two-tailed).

### 4.4 Structural model of perceived teacher emotional support, academic burnout, and online learning engagement

The structural equation model, with perceived teacher emotional support as the independent variable, academic burnout as the mediating variable, and student online learning engagement as the dependent variable, is illustrated in the figure. After model modification, the model demonstrated a good fit, with the following fit indices: CMIN/DF = 1.587 < 3, GFI = 0.97 > 0.90, CFI = 0.989 > 0.90, NFI = 0.971 > 0.90, IFI = 0.989 > 0.90, SRMR = 0.052 < 0.08, and RMSEA = 0.053 < 0.08.

**Figure 1:**
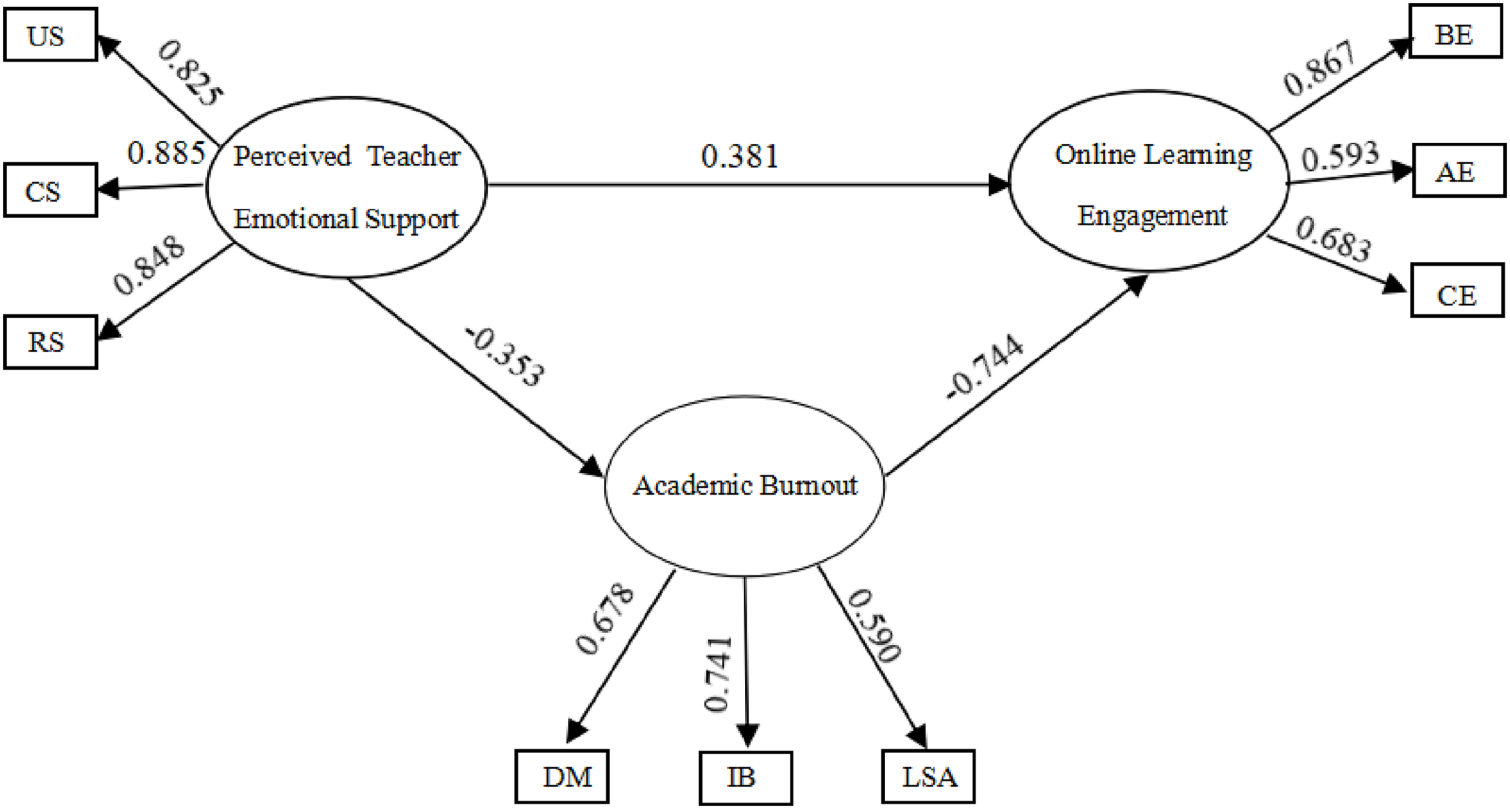
Structural model of perceived teacher emotional support, academic burnout, and online learning engagement.

As shown in Table 4 and Figure 1, perceived teacher emotional support significantly and positively predicts online learning engagement, with a direct effect value of 0.407 (*p* < 0.001), supporting Hypothesis H1. Furthermore, perceived teacher emotional support also negatively influences online learning engagement through academic burnout, supporting Hypothesis H2 and H3, and indicating a significant mediating effect of academic burnout in the relationship between perceived teacher emotional support and online learning engagement, with a mediating effect value of 0.281 (*p* < 0.001).

## 5 Discussion

This study aims to examine the status of university students’ online learning engagement, perceived teacher emotional support, and the level of academic burnout during online learning. Additionally, it seeks to verify the mediating role of academic burnout in the relationship between perceived teacher emotional support and online learning engagement.

The findings showed that the level of perceived teacher emotional support was moderate to high, which aligns with the results of He et al. (2024). This suggests that, during online learning, university students generally perceive teacher emotional support well. Additionally, no significant gender differences were found in the perception of teacher emotional support. In this study, university students’ online learning engagement was found to be at a moderate level, with an average score ranging from 3 to 3.5. This result is consistent with the findings of Wang L. (2022). This suggests that while students participate in various learning activities and complete learning tasks during online learning, they are not highly proactive. Overall, the level of online learning engagement among university students is not ideal.

Furthermore, the study confirmed that perceived teacher emotional support significantly predicts online learning engagement. This finding is consistent with previous research by Lobo (2023) and Lavy and Naama-Ghanayim (2020). Their research also demonstrated that perceived teacher emotional support greatly enhances meaningful learning engagement.

Based on the direct effect size and the proportion of the total effect of perceived teacher emotional support on online learning engagement (0.407, 59.2%), it is evident that perceived teacher emotional support exerts a stronger impact on students’ engagement in online learning. The scale results indicate that all three dimensions of perceived teacher emotional support have a significant impact on learning engagement, primarily reflected in the respect and care for students’ autonomy. Compared to primary and secondary school students, university students exhibit greater autonomy and individuality, requiring teachers to provide more respect and support for their ideas and actions. This is consistent with the work of Ruzek et al. (2016), who found that teachers’ emotional support motivates students through their perceived autonomy and competence. Additionally, perceived teacher emotional support plays a crucial role in helping students cope with the difficulties encountered in online learning. Since online learning imposes certain demands on the learning environment, internet connectivity, and devices, it can create additional pressure on students. Therefore, perceived teacher understanding and assistance become even more important. With sufficient emotional support, university students experience a sense of love and security, fulfilling their psychological needs and making them more willing to engage in learning, as noted by Jin and Wang (2019).

This study found that university students’ academic burnout was at a moderate level, with no significant gender differences. The lowest average score was in the “low achievement” dimension, while the highest was in the “emotional exhaustion” dimension. These findings are consistent with research by Zhao et al. (2018). The study also confirmed that perceived teacher emotional support is negatively correlated with academic burnout, which aligns with the results of Huang et al. (2024) and Li and Zhang (2024). Previous studies have consistently shown that in online learning environments, perceived teacher emotional support can alleviate academic burnout among university students.

The underlying reason is that among the factors contributing to academic burnout, the depressed mood dimension exhibits the highest factor loading, making it the dominant factor. In the online learning environment, prolonged study sessions increase feelings of burnout, frustration, and fatigue. However, teachers can foster emotional presence through synchronous interactions and asynchronous feedback, such as “paying attention to my learning progress” and “understanding my negative emotions.” These actions help students feel respected, cared for, and understood, which alleviates negative emotions and boosts academic engagement. These findings align with the research of He et al. (2024).

This study reveals the relationships among perceived teacher emotional support, academic burnout, and university students’ learning engagement. It confirms the positive effects of perceived teacher emotional support in reducing academic burnout and enhancing online learning engagement, offering important implications for online teaching and learning.

First, in the online learning environment, teachers should prioritize building strong teacher-student relationships to encourage students to open up, thereby enhancing their perception of teacher emotional support (Akram and Li, 2024). For instance, since a teacher’s image is conveyed to students through digital media, attention should be given to language expression, body language, and facial expressions to project warmth and approachability. Teachers can also engage in regular in-depth communication with students and provide timely feedback. Through positive feedback, students can feel valued and acknowledged. Additionally, as online learning may lead to increased feelings of loneliness and anxiety, teachers should actively show concern for students’ learning progress and overall well-being, offer praise for their achievements, and provide encouragement in times of difficulty to strengthen the teacher-student bond.

Second, teachers should focus on enhancing students’ sense of academic achievement to alleviate academic burnout. This can be achieved by leveraging online platforms to recognize and validate students’ learning outcomes in real time. For example, publicly praising students for insightful contributions in discussion forums or outstanding performance on assignments can help them clearly see the results of their efforts, thereby boosting their sense of accomplishment. Additionally, assigning challenging learning tasks can further enhance students’ achievement motivation. When students successfully complete such tasks, their sense of achievement increases, fostering intrinsic motivation and encouraging active engagement in learning, ultimately reducing academic burnout.

Third, teachers should pay close attention to students’ negative emotions in online learning and take proactive measures to prevent academic burnout (Wang et al., 2024). Creating a harmonious and positive online learning atmosphere allows students to study in a relaxed and enjoyable environment, making them more willing to participate in learning activities, collaborate with peers, and enhance their learning enthusiasm and initiative. Furthermore, providing individualized emotional support based on students’ unique needs can strengthen their psychological resilience, enabling them to approach challenges with a more positive mindset, thereby effectively preventing academic burnout.

## 6 Conclusion

This study provides an in-depth examination of the impact of perceived teacher emotional support on online learning engagement and validates the mediating role of academic burnout. The main findings are as follows:

First, the study reveals that university students’ perceived teacher emotional support, academic burnout, and learning engagement during online learning are all at a moderate level, with no significant gender differences. Additionally, our findings highlight the critical role of perceived teacher emotional support in enhancing online learning affective, significantly influencing behavioral, cognitive, and affective engagement.

Second, academic burnout serves as a key mediating factor affecting learning engagement in online learning environments. Perceived teacher emotional support not only directly enhances online learning engagement but also exerts an indirect effect by alleviating academic burnout, further strengthening engagement.

Despite these findings, the study has several limitations. First, the data for the study variables were primarily collected through self-reported scale, which may introduce subjectivity and limit the ability to accurately reflect objective realities. Second, this study focused exclusively on first-year students majoring in computer-related disciplines. Due to the gender imbalance within this major, the generalizability of the findings may be limited. Future research will aim to expand the sample size to include students from different academic years and cultural backgrounds, in order to validate the conclusions. Finally, this study focuses solely on the positive effects of perceived teacher emotional support on academic burnout and learning engagement. Future research should consider incorporating teacher autonomy support and cognitive support into the framework to comprehensively examine the mechanisms through which teacher support influences learning engagement.

## Data Availability

The original contributions presented in the study are included in the article/supplementary material, further inquiries can be directed to the corresponding author.
